# Innovation in hepatic alveolar echinococcosis imaging: best use of old tools, and necessary evaluation of new ones[Fn FN1]

**DOI:** 10.1051/parasite/2014072

**Published:** 2014-12-23

**Authors:** Wenya Liu, Éric Delabrousse, Oleg Blagosklonov, Jing Wang, Hongchun Zeng, Yi Jiang, Jian Wang, Yongde Qin, Dominique Angèle Vuitton, Hao Wen

**Affiliations:** 1 Imaging Center, First Affiliated Hospital, Xinjiang Medical University Hospital No. 1 Liyushan road Urumqi 830011 PR China; 2 Department of Visceral Radiology, University Hospital Jean Minjoz 25030 Besançon France; 3 WHO-Collaborating Centre for Prevention and Treatment of Human Echinococcosis, University of Franche-Comté and University Hospital 25030 Besançon France; 4 Department of Nuclear Medicine, University Hospital Jean Minjoz 25030 Besançon France; 5 Department of Ultrasonography, First Affiliated Hospital, Xinjiang Medical University Hospital No. 1 Liyushan road Urumqi 830011 China; 6 Department of Hepatic surgery, First Affiliated Hospital, Xinjiang Medical University Hospital No. 1 Liyushan road Urumqi 830011 China

**Keywords:** Hepatic Alveolar Echinococcosis, Computed Tomography, Magnetic Resonance Imaging, FluoroDeoxyGlucose-Positron Emission Tomography, Functional evaluation, Innovation techniques

## Abstract

Hepatic Alveolar Echinococcosis (HAE), caused by larvae of *Echinococcus multilocularis*, is a rare but potentially lethal parasitic disease. The first diagnostic suspicion is usually based on hepatic ultrasound exam performed because of abdominal symptoms or in the context of a general checkup; HAE diagnosis may thus also be an incidental finding on imaging. The next step should be Computed Tomography (CT) or Magnetic Resonance Imaging (MRI). They play an important role in the initial assessment of the disease; with chest and brain imaging, they are necessary to assess the PNM stage (parasite lesion, neighboring organ invasion, metastases) of a patient with AE. Performed at least yearly, they also represent key exams for long-term follow-up after therapeutic interventions. Familiarity of radiologists with HAE imaging findings, especially in the endemic regions, will enable earlier diagnosis and more effective treatment. Fluorodeoxyglucose Positron Emission Tomography (FDG-PET) is currently considered to be the only noninvasive, albeit indirect, tool for the detection of metabolic activity in AE. Delayed acquisition of images (3 hrs after FDG injection) enhances its sensitivity for the assessment of lesion metabolism and its reliability for the continuation/withdrawal of anti-parasite treatment. However, sophisticated equipment and high cost widely limit PET/CT use for routine evaluation. Preliminary studies show that new techniques, such as contrast-enhanced ultrasound (US), Dual Energy CT or Spectral CT, and Diffusion-Weighted MRI, might also be useful in detecting the blood supply and metabolism of lesions. However, they cannot be recommended before further evaluation of their reliability in a larger number of patients with a variety of locations and stages of AE lesions.

## Abstract (fr)

L’échinococcose alvéolaire hépatique (EAH), causée par les larves d’*Echinococcus multilocularis*, est une maladie parasitaire rare mais potentiellement fatale. La première suspicion diagnostique provient généralement d’une échographie hépatique réalisée en raison de symptômes abdominaux, ou à l’occasion d’un bilan plus général; la découverte de l’EAH est donc parfois fortuite. L’étape suivante doit comporter un scanner et/ou une imagerie en résonance magnétique nucléaire (IRM), qui, incluant la recherche de localisations pulmonaires et cérébrales, jouent un rôle important dans l’évaluation initiale de la maladie et sont absolument nécessaires pour déterminer le stade PNM (lésion parasitaire, invasion des organes proches, métastases) de l’EA. Répétées au moins une fois par an, elles représentent les examens-clés pour le suivi des patients à long-terme et le management de leur traitement. Une meilleure familiarité des radiologues avec l’imagerie de l’EAH contribuerait largement au diagnostic précoce de l’affection dans les zones d’endémie et à un traitement plus efficace. La Tomographie par Émission de Positons au Fluorodéoxyglucose (FDG-TEP) est actuellement considérée comme la meilleure technique non-invasive, bien qu’indirecte, d’évaluation métabolique de l’EAH. L’acquisition tardive (3 heures après injection du FDG) des images améliore sa sensibilité diagnostique et sa fiabilité comme indicateur de poursuite/arrêt du traitement antiparasitaire. Cependant, l’équipement sophistiqué et le coût élevé de cette technique en limite considérablement l’utilisation en routine. Les résultats préliminaires obtenus avec des techniques plus abordables d’évaluation fonctionnelle comme l’échographie de contraste, le scanner en acquisition spectrale ou bi-énergie, ou l’IRM pondérée en diffusion, suggèrent qu’elles sont capables d’évaluer aussi la suppléance vasculaire et l’activité métabolique dans cette indication. Elles ne peuvent cependant pas être recommandées pour une utilisation de routine avant que des études sur un nombre de patients plus élevé, présentant des localisations et des stades évolutifs variés, n’aient été réalisées.

## Abstract (zh)

肝泡型包虫病是一种少见的但具有致命风险的寄生虫病。其初诊往往基于患者出现腹部不适症状或常规体检时所进行的超声检查；也就是说肝泡型包虫病多数情况是在影像学检查时偶然发现的。接下来这些患者就要进行肝脏的CT或磁共振（MRI）扫描 。这两项检查对本病的初步评估起着重要作用，头颅、胸部的扫描也必须同时进行以便进行 PNM分期。CT和MR在治疗后的随访工作中仍起着关键作用，建议至少一年进行一次随访检查。对泡型包虫病影像诊断富有经验的放射科医师，尤其是那些在疾病流行区域的医师，往往能够对本病进行早期诊断并指导更有效的治疗。氟脱氧葡萄糖标记的正电子发射断层成像（FDG-PET）是目前所公认唯一的一种能以间接无创的方式评价泡型包虫代谢活性的检测工具。FDG-PET延迟显像（在注射FDG3小时后）对判断包虫病灶代谢活性敏感并且能够为决定是否停用化疗药物提供有力的依据。然而PET/CT这种高端设备以其高昂的检查费用使它不能够普及应用来进行泡型包虫病的评估。初步研究表明，新的成像技术如超声造影、双能/能谱CT和磁共振弥散加权成像，有望能够检测病变的血供情况及判断代谢活性。然而，这些成像方法目前还不能推广，尚需进行多中心大样本和不同分期病例的进一观察期来确定其临床应用的可靠性。

## 1. Introduction

Hepatic Alveolar Echinococcosis (HAE) caused by the larvae (metacestode) of *Echinococcus multilocularis* is a rare disease only present in the northern hemisphere, compared with Cystic Echinococcosis (CE) caused by *Echinococcus granulosus*, which is present worldwide. Although histopathologically a benign disease, due to a parasite, HAE shows the characteristics of a malignant tumor with destructive tissue growth, invasion of adjacent organs, and distant dissemination [12, 31].

Because HAE is a tumor-like and chronic disease, with a latent stage that may last for years before signs and symptoms develop, the diagnosis primarily depends on imaging techniques including ultrasound (US), computed tomography (CT), and magnetic resonance imaging (MRI) [4, 32]. Although HAE is still often diagnosed on clinical symptoms in low-resource countries, including China where the disease is endemic in rather remote areas of the western provinces and autonomous regions [23], it is more and more often disclosed incidentally on imaging in Europe, the second major endemic area for this disease [47]. Depending on the stage when they are discovered and their location, there is a spectrum of possible images that are often characteristic of the disease but may nevertheless lead to difficulties in differential diagnosis. Imaging techniques are also used for the follow-up of patients, with the aim of evaluating the efficacy of the treatment (surgery and chemotherapy using albendazole) and the viability of the parasite. Ultrasound and the conventional radiological imaging techniques are not well adapted to this purpose, and Positron Emission Tomography (PET) with [18F]fluorodeoxyglucose (FDG) as a radioisotope tracer has been proposed for such an assessment [37]. More sophisticated imaging techniques, such as Contrast-Enhanced Ultrasound (CEUS), dual energy CT and spectral CT, or MR-Diffusion-Weighted Imaging (DWI), have become available; some of them could be promising in their applications to HAE. In addition, whatever the technique used to obtain images of AE lesions, their correlation with the underlying complex pathology of HAE has very rarely been studied. However, more precise delineation of the pathological “substratum” of the images obtained with the various available techniques would be important to better interpret imaging aspects. This review will focus on the images obtained from well-validated imaging techniques in HAE and when each technique should be used for diagnosis and follow-up. It will then describe the images obtained with newer imaging procedures recently introduced and try to delineate their pathological counterpart. Whenever possible, it will tentatively establish their additional value, if any, for diagnosis and follow-up.

## 2. Pathology of HAE

Humans are accidentally infected with *E. multilocularis* by ingestion of eggs dispersed in soil, vegetables, and animal fur, and by the feces of carnivores (foxes and dogs) harboring adult tapeworms of this species. Oncospheres hatched from eggs in the small intestine of humans migrate via the portal system to the liver, where they differentiate and develop into the metacestode stage. The larva causes invasive and destructive changes in the liver and behaves like a malignant neoplasm. Since clinical symptoms usually do not become evident until 10 or more years after initial parasite infection, early diagnosis and treatment especially during the asymptomatic period are important for the reduction of morbidity and mortality [8].

The metacestodes propagate asexually like a tumor, by buds then vesicles, leading eventually to bile duct and hepatic vessel obstruction and organ dysfunction. Cellular immune suppression of the host leads to faster growth both in experimental animals and in humans [2]. At gross pathologic analysis, the HAE lesion appears as a whitish infiltrative multivesicular mass composed of many cysts irregular in shape and size (diameters from <1 to 20 mm) with associated fibrosis and calcification. There is no clear margin between the parasitic tissue and the adjacent normal liver parenchyma. In the human host, both metacestode proliferation and cellular host response may produce lesions up to 15–20 cm in diameter. *E. multilocularis* actually produces multiple vesicles (small cysts of 1–20 mm in diameter) that resemble alveoli, hence the name of the disease, and give a “multilocular” aspect to the lesions, hence the name of the cestode. The metacestode grows by exogenous proliferation, with the vesicles progressively invading the host tissue by peripheral extension of the process originating in the “germinal layer”, each vesicle being rapidly surrounded by a polysaccharide-rich “laminated layer” [19]. From the very beginning of larval proliferation too, the immune response of the host is characterized by the homing to the liver of cells such as macrophages, lymphocytes, fibroblasts, and myofibroblasts, at the contact of the laminated layer, and which display a “granulomatous” structure [20]. Cytokines and chemokines are major actors in cell homing, as well as in cell functions of the periparasitic infiltrate [48]. Fibrosis and necrosis, which tend to limit the extension of the metacestode, are the main functional consequences of periparasitic cell activation. The periparasitic cell infiltrate is usually located at the periphery of the lesions, in areas with active proliferation of the metacestode [6]. Neo-vessels accompany immune cell homing and are present in this “granulomatous” part of the lesion [21, 45, 46]. Necrotic areas, conversely, are usually located in the most inactive part of the lesion, where dead vesicles and “giant cells”, i.e. multinuclear scavenger macrophages, are present. Those necrotic cavities developed in the degenerating areas of the metacestode, which may reach sizes up to tens of centimeters, are in no way an equivalent of CE “cysts”. This is why the term “pseudo-cyst” and not “cyst” will always be used in this review to designate these necrotic areas. The word “vesicles” or “cysts” will be restricted to the elementary structure defined by the germinal and laminated layers of the metacestode (i.e. the active, proliferating, parasite unit). As the lesion heals, it invariably becomes calcified; from punctuate to multiple scattered and peripheral calcifications, and/or sometimes a large homogeneously calcified mass [39]. Progressive obstruction of portal veins or their branches as well as a direct proliferative influence of the metacestode on normal hepatocytes are responsible for liver regeneration in those segments/lobes of the liver not involved in parasitic growth [28, 53].

## 3. Imaging findings in HAE using well-validated techniques

Imaging is the major method used for the diagnosis of HAE, while serological tests are used to confirm diagnostic suspicion raised by imaging observations [16]. In general, US is the initial investigative modality of choice for the detection of HAE, including for mass screening [3, 11, 29] and is usually complemented by CT scans which best show the calcifications that are characteristic of HAE. CT is also able to comprehensively depict the morphological changes in the liver of HAE patients. Though CT is the second preferred imaging exam, MRI is also used in combination with US when HAE involves the biliary tree, and/or to disclose the multivesicular pathognomonic structure of the lesion, and thus ascertain AE diagnosis.

### 3.1. Ultrasonography (US): the imaging tool of choice for the detection of AE lesions

**Figure 1.**
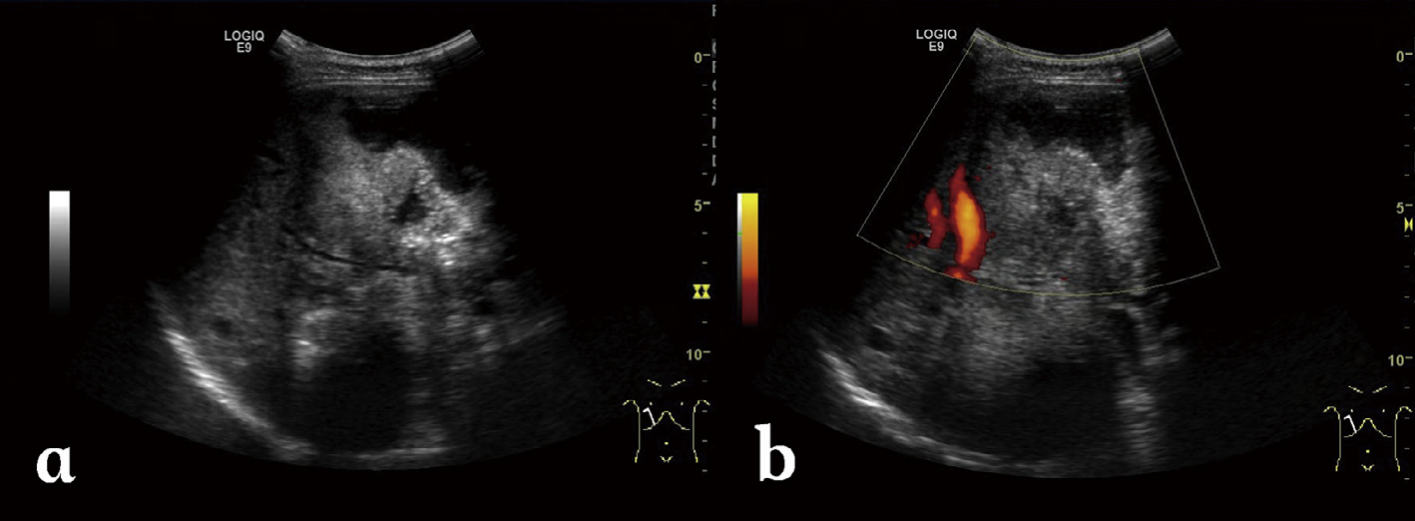
*Alveolar echinococcosis in a 30-year-old woman.* (a) Abdominal gray-scale US image shows an irregular type heterogeneous mass lesion with no clear boundary in the right lobe of the liver, containing anechoic pseudo-cystic lesion and hyperechoic foci of calcification. (b) Color Doppler US image shows no obvious blood flow signal while cystic duct is constricted.

Typical findings in abdominal US include a hepatic mass with mixed hyper- and hypoechogenicity, irregular margins, scattered foci of calcification, and a pseudo-cyst which is caused by the central necrosis surrounded by an irregular ring-like hyperechogenicity region which represents fibrous tissue (Fig. 1). Less typical appearances include multiple clustered hemangioma-like hyperechoic nodules (referred to as “hailstorm pattern”), small calcified lesions, and apparently isolated pseudo-cysts with massive necrosis. In the presence of the latter, the differential diagnosis includes benign liver cyst, cystadenoma, cystadenocarcinoma, and *E. granulosus* lesions. However, irregular borders and a lack of enhancement are suggestive features of HAE; the other lesions usually exhibit peripheral enhancement and are rarely calcified. A pseudo-cystic appearance may also be seen in recurrent foci of HAE after percutaneous drainage of primary lesions.

Color and pulsed Doppler US images can show distortion and displacement of the hepatic veins, portal vein, and biliary tree resulting from a mass effect, invasion of the inferior vena cava or hepatic or portal vein walls, and secondary biliary duct involvement and intrahepatic bile duct dilation [3, 7, 8].

US imaging is the first choice for screening and follow-up due to its widespread availability, radiation-free method, and low costs. Because of its limited contribution to the identification of small peripheral lesions, unsatisfactory evaluation of the extension into the perihepatic adipose tissue and neighboring organs, and occasionally insufficient detection of small-sized (<2 cm) lesions containing calcifications, other imaging techniques, whenever available, should be performed for better evaluation.

### 3.2. Computed Tomography (CT): for better characterization of AE lesions and their typical calcifications

**Figure 2.**
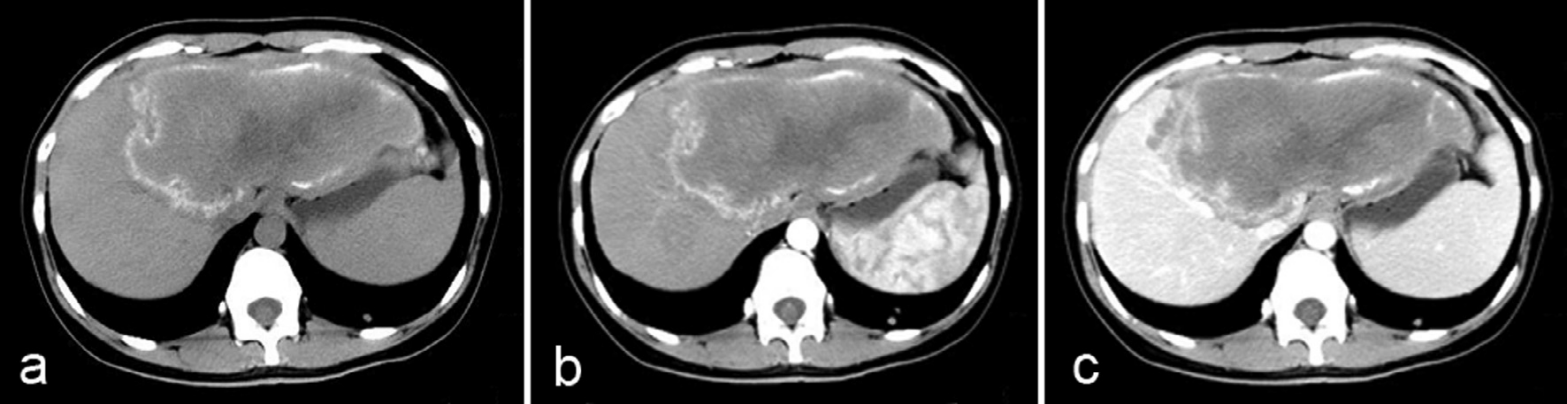
*Alveolar echinococcosis in a 28-year-old woman.* (a) Axial unenhanced CT image shows an infiltrative tumor-like hepatic mass involving both lobes of the liver with irregular margin and heterogeneous contents including hyper-attenuating foci of calcification scattered peripherally and an area of hypo-attenuation corresponding to necrosis and parasitic tissue centrally. (b) Post-contrast arterial phase shows a non-enhancing, hypo-attenuating lesion. (c) Post-contrast portal-venous phase shows a faint enhancement of fibro-inflammatory components surrounding the parasitic pseudo-cyst.

Unenhanced CT images show an infiltrating tumor-like hepatic mass with irregular margins and heterogeneous contents with varied attenuation, including scattered hyper-attenuating calcifications and hypo-attenuating areas corresponding to necrosis (Fig. 2a). No substantial enhancement is observed within the hepatic lesion after administration of an intravenous contrast medium (Fig. 2b); however, the fibro-inflammatory component surrounding the parasitic tissue may be enhanced faintly in the delayed phase after injection (Fig. 2c). In pseudo-cystic hepatic lesions, CT shows a large necrotic central area surrounded by an irregular ring-like region of fibrous tissue that is often partly calcified. If the parasitic process has infiltrated the hilum, the intrahepatic bile ducts typically appear dilated. Hepatomegaly of the contralateral “healthy” liver lobe is often conspicuous.

**Figure 3.**
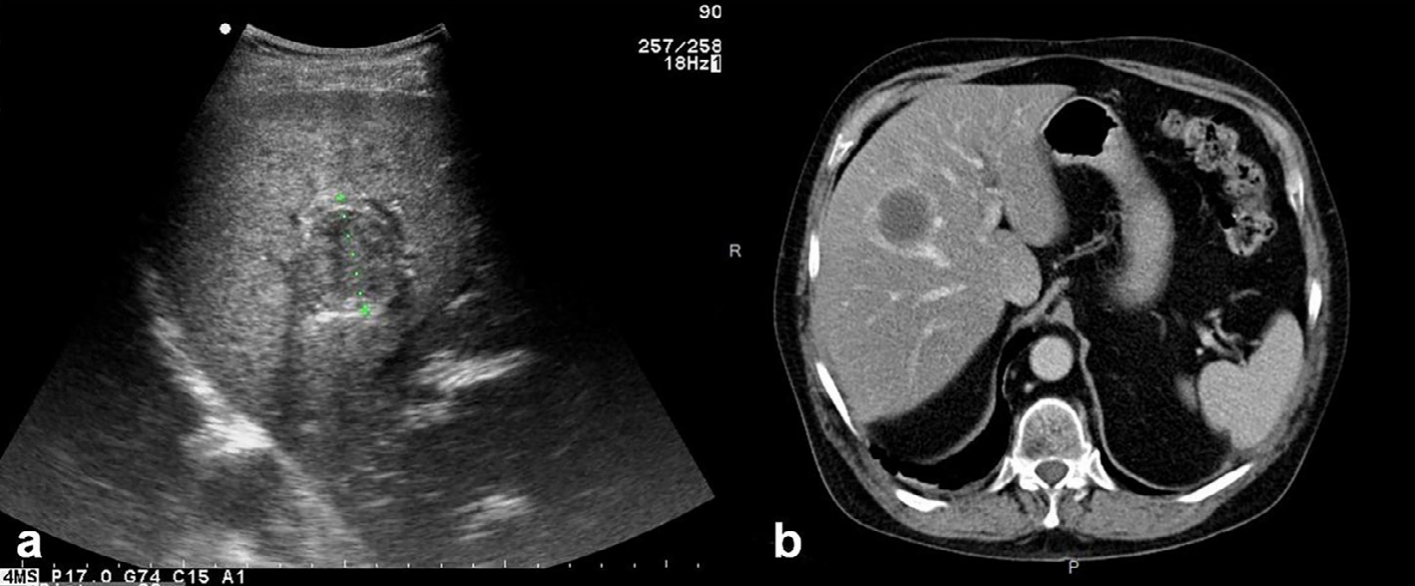
*Alveolar echinococcosis in a patient with immunosuppression.* (a) Abdominal gray-scale US shows an abscess-like hepatic image. (b) Axial unenhanced CT shows a typical aspect of pyogenic liver abscess.

CT features of HAE lesions have been reported to be a differential diagnosis for primary hepatic neoplasms such as cholangiocarcinoma, biliary cystadenoma, and biliary cystadenocarcinoma, as well as for hepatic metastases. However, the CT findings of hypo-attenuation, calcification, and absence of contrast enhancement in a hepatic lesion usually help identify it as HAE [22, 25]. Recent observations of liver metastasis-like images in patients with digestive cancer, eventually leading to the discovery of HAE after months of misdiagnosis, have however dampened the notion of a usually easy differential diagnosis [10]; so have hemangioma-like and liver abscess-like images in patients with acquired immune suppression due to chemotherapy and/or anti-TNF biotherapeutic agents (Fig. 3) [50]. Such images, rarely described in the past, could be due to the early disclosure of the lesions and/or to the rapid growth of the metacestode.

Though radiation is the main drawback of CT scan, CT remains the mainstream modality for morphologic imaging assessment of HAE lesions in the majority of low-resource countries or districts because CT has a clear superiority over MR in demonstrating calcifications, especially in small clusters. Better than US, it helps to determine the number, size, and location of lesions in the liver and allows a comprehensive preoperative evaluation of vascular, biliary, and extrahepatic extension, which is an important consideration when assigning a stage to the patient in the PNM (parasite lesion, neighboring organ invasion, metastases) classification (see below and Table 1) and in assessing the possibility of resection of the HAE.

**Table 1.** PNM classification of alveolar echinococcosis (according to WHO-IWGE, 2006).

PNM	Location and extension of the lesion
**P**	Hepatic localization of the parasite
P X	Primary parasite cannot be assessed
P 0	No detectable parasite in the liver
P 1	Peripheral lesions without proximal vascular and/or biliary involvement
P 2	Central lesions with proximal vascular and/or biliary involvement of one lobe^a^
P 3	Central lesions with hilar, vascular, or biliary involvement of both lobes and/or with involvement of two hepatic veins
P 4	Any liver lesion with extension along the vessels^b^ and the biliary tree
**N**	Extrahepatic involvement of neighboring organs [diaphragm, lungs, pleura, pericardium, heart, gastric and duodenal wall, adrenal glands, peritoneum, retroperitoneum, parietal wall (muscles, skin, bone), pancreas, regional lymph nodes, liver ligaments, kidneys]
N X	Not evaluable
N 0	No regional involvement
N 1	Regional involvement of contiguous organs or tissues
**M**	The absence or presence of distant Metastasis [lungs, distant lymph nodes, spleen, CNS, orbital, bones, skin, muscle, kidneys, distant peritoneum, and retroperitoneum]
M X	Not completely evaluated
M 0	No metastasis^c^
M 1	Metastasis

a For classification, the plane projecting between the bed of the gall bladder and the inferior vena cava divides the liver into two lobes.

b Vessels mean inferior vena cava, portal vein, and arteries.

c Chest X-ray and cerebral CT negative.

### 3.3. Magnetic Resonance Imaging: to disclose pathognomonic images and perform a pre-therapeutic assessment

With high resolution for soft tissue and without any radiation, MR imaging is the best modality for characterizing the components of parasitic lesions and depicting vascular or biliary tree involvement and extrahepatic extension [4, 25].

**Figure 4.**
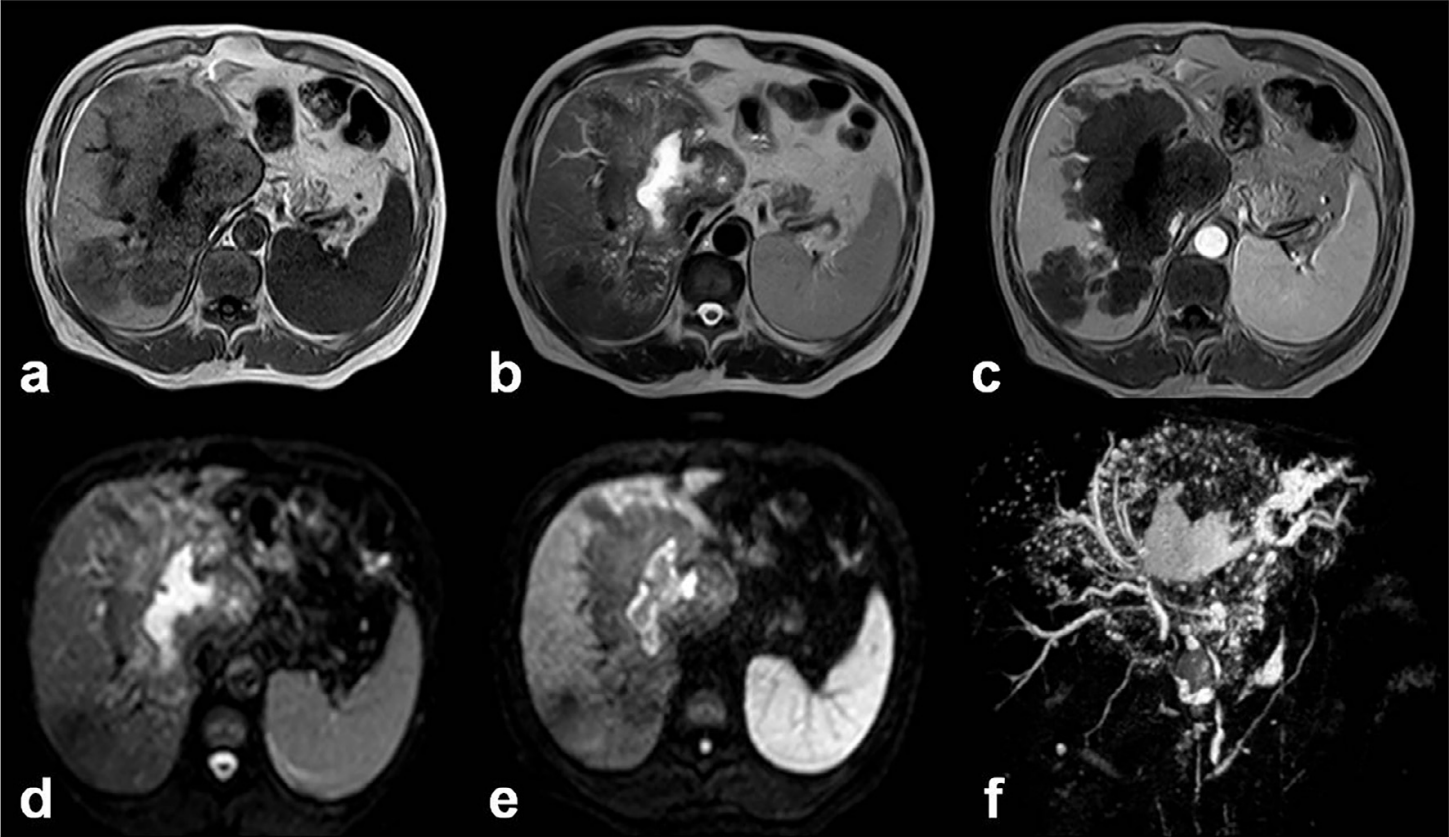
*Alveolar echinococcosis in a 55-year-old man.* (a) Axial unenhanced T1-weighted Magnetic Resonance (MR) image shows infiltrating diffused mass with hypo-intense signal in the right and left lobes of the liver. (b) Axial unenhanced T2-weighted MR image shows heterogeneous signal. The high signal intensity corresponds to small cystic and necrotic components of mass lesion. (c) Axial enhanced T1-weighted image with gadolinium shows no enhancement in the mass lesion. (d, e) Diffusion-weighted MR images obtained with b value of 0 s/mm^2^ and 600 s/mm^2^. Hypo-intense signal characterizes the cystic and necrotic components of the lesion whereas hyper-intense signal seen in the central necrotic part reveals restricted diffusion due to bacterial superinfection. (f) MR Cholangiopancreatography of the liver shows several small cystic and necrotic components of the mass lesion with dilated right hepatic duct, constricted upper part of common bile duct, and mildly dilated pancreatic duct.

Characteristic MR imaging features of HAE at the middle/late stage include a heterogeneous infiltrative mass with irregular margins and a necrotic center that exhibits low to intermediate signal intensity on T1-weighted images and heterogeneous signal intensity (areas of low and high signal intensity) on T2-weighted images (Figs. 4a and 4b). Areas of high T2 signal intensity correspond to small cystic or necrotic components, whereas areas of low T2 signal intensity correspond to fibrotic or collagenous components. T2-weighted images are useful for detecting small hepatic and extrahepatic vesicles and their typical arrangement. The multivesicular structure of AE lesions, with nearly pathognomonic features of “honeycomb” or “bunch of grapes”, is best disclosed on MRI T2-weighted images which thus represent the best confirmation imaging exam in case of difficult diagnosis. Kodama et al. [25], from a series of AE patients living in Hokkaido, Japan, proposed in 2003 a classification of MR images in HAE, with five classes as follows: type 1: multiple small round cysts without a solid component; type 2: multiple small round cysts with a solid component; type 3: a solid component surrounding a large and/or irregular pseudo-cyst with multiple small round cysts; type 4: a solid component without cysts; type 5: a large cyst without a solid component. On a series of French AE patients, we studied the FDG-PET/CT characterization of the lesions defined according to this MRI classification and could show that the presence of vesicles/small cysts was significantly associated with positive images on FDG-PET/CT [1]. This observation strongly suggests that disclosure of micro-cystic images on MRI could be a good surrogate marker of parasite viability; however, such findings should be confirmed by prospective studies in a higher number of HAE cases [1].

MR cholangiopancreatography (MRCP) yields information about the relationship between HAE lesions and the biliary tree; in particular, it can depict biliary dilation, a reduced number of bile ducts within the lesion, invasion of the biliary wall, distortion and compression of the biliary tree, and communication of intrahepatic bile ducts with necrotic pseudo-cystic regions (Fig. 4f). Such pieces of information are precious before any decision on radical surgical resection, and before percutaneous or perendoscopic interventional palliative procedures [4].

### 3.4. Positron Emission Tomography (PET): an imaging tool for patient follow-up

**Figure 5.**
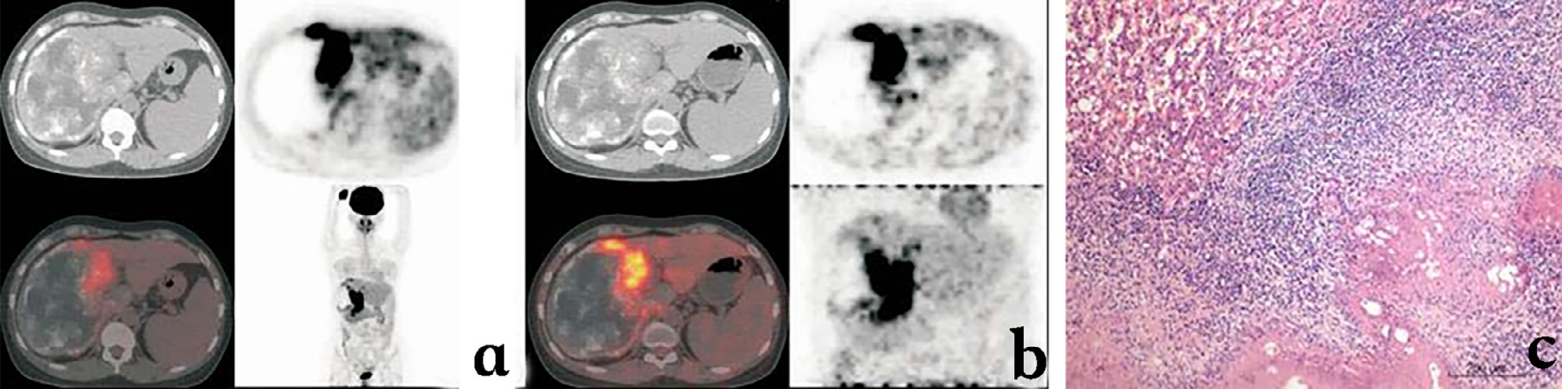
*18F-Fluorodeoxyglucose (FDG) Positron-emission tomography (PET) aspects of hepatic alveolar echinococcosis.* (a) Early image acquisition (1 hour after FDG injection): large-sized heterogeneous space-occupying lesion in the right lobe of the liver, with multiple scattered calcifications and areas of liquefaction necrosis, without obvious FDG uptake inside the lesion; significant FDG uptake at the border of the lesion, at the junction of the right and left lobes, irregular in shape. (b) Delayed image acquisition (3 hours after FDG injection): significantly enhanced images of FDG uptake, with an increase in SUV value. (c) Pathology of the HAE lesion sampled in the area of high FDG uptake: fibroblast proliferation, associated with macrophage, lymphocyte, plasma cell, and eosinophil-rich inflammatory infiltrate.

**Figure 6.**
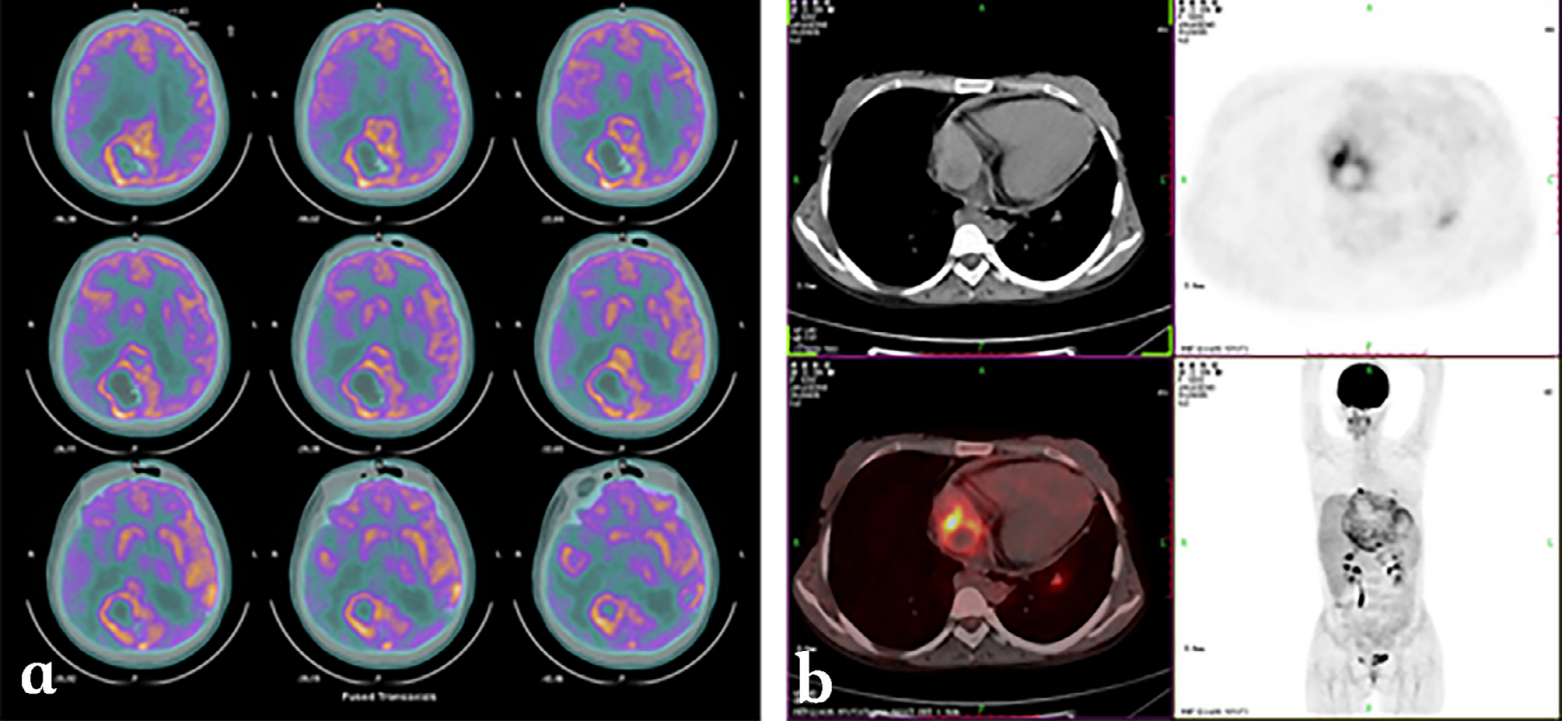
*18F-Fluorodeoxyglucose (FDG) Positron-emission tomography (PET) aspects of extrahepatic alveolar echinococcosis.* (a) Brain metastasis of alveolar echinococcosis. (b) Right atrium metastasis of alveolar echinococcosis.

Information on parasite metabolic activity cannot be obtained by conventional imaging techniques. In addition, changes in the size of the lesions after albendazole treatment are slow and US as well as CT and MRI may, at best, evaluate absence of progression rather than regression of lesions in most cases of HAE; decrease in the size of pseudo-cysts after drainage is in no way evidence of lesion regression, but may be of help to reassess the technical possibility of lesion resection by surgery [4]. [18F]FDG-PET has been proposed as a non-invasive tool for the three-dimensional detection of metabolic activity in HAE lesions [13, 37]. Location of FDG uptake within HAE lesions, at the periphery of the lesions [40], as well as in vitro experiments which have shown high uptake of this radioisotope tracer by immune cells and rather low uptake by *E. multiloculari*s cells/vesicles [33], strongly suggest that FDG-PET does not evaluate parasite viability/lesion progression directly (Fig. 5). Increased glucose metabolism in cells of the host’s response is very likely responsible for PET-positive images in HAE, which thus only indirectly assess disease progression [36]. As it explores only a part of the lesions and the images are not specific to AE, PET/CT cannot be used as a diagnostic tool in HAE. Nevertheless, as there is currently no imaging tool better adapted to the follow-up of patients with HAE, whenever possible, initial assessment of the metabolic activity of the lesions by FDG-PET/CT before the initiation of any treatment may be recommended, as a basis for further follow-up. However, at Besançon University Hospital, we have demonstrated that delayed acquisition of the images, i.e. measuring FDG uptake 3 hrs after injection and not only 1 hr after injection as usually performed in oncology, was necessary to improve the sensitivity of the procedure, and reduce the risk of false-negative images [9]. With this precaution, in patients with radical operations, PET/CT may help detect early hepatic relapses and allow timely intervention. In patients treated with albendazole alone or combined with interventions aimed at alleviating complications, retrospective studies have shown that, albeit more expensive than conventional imaging techniques, FDG-PET might prove cost-effective by permitting the duration of chemotherapy to be shortened [35]. Recurrence of the disease after albendazole withdrawal has however been observed in patients with negative PET/CT using standard acquisition of images [35]; this further supports the interest of late acquisition of images in HAE [36]. Prospective evaluation is currently ongoing to delineate usefulness and best modalities of PET/CT follow-up to monitor disease activity, to optimize therapeutic management, and, as a final goal, to reassess long-term treatment with benzimidazoles on the basis of clearly discriminating imaging findings [44]. This is among the objectives of the EchinoVista project in France [5]. Finally, the use of FDG-PET/CT to disclose and monitor extrahepatic locations of AE remains questionable: increased FDG uptake by lung, heart, and brain metastases has been observed in Chinese patients with advanced lesions (Fig. 6); such an uptake is observed far more rarely in French patients with smaller and/or less advanced lesions (O Blagosklonov, personal observations); this actually warrants further systematic evaluation in a higher number of AE patients with AE metastases in various locations, at various stages, and in patients with and without immunosuppression.

## 4. Staging of HAE

Staging of AE is the process of determining how much larval tissue there is in the body and where it is located. In addition, staging has a prognostic objective, since it describes the extent and location of an individual’s hepatic lesion based on its proximity to bile ducts and hepatic vessels, and the extent of its spread, locally and distantly in the body. Such a staging system was designed by the WHO-Informal Working Group on Echinococcosis, on the model of the “TNM” classification and staging of tumors, to be simply applicable in different settings (Table 1). It aims at facilitating communication among clinicians, providing guidance for the most appropriate treatment strategy, and gaining standardized information on the course and outcome of the disease. In addition, determining the stage can also help to identify patients that could be recruited for multicenter trials [24].

The stage of AE is based on three main factors: (1) Location and extension of the primary (original) parasitic lesion within the liver (P); (2) Involvement of neighboring organs (whether or not the larva has spread to the nearby tissues/organs, including lymph nodes) (N); (3) Presence or absence of metastasis (whether or not the larva has spread to distant areas of the body, such as the lungs, brain, bones, or any other location) (M).

The overall purpose of the PNM classification is to improve the quality of treatment and allow uniform evaluation of outcomes across healthcare institutions [24, 43]. Results obtained by the various imaging techniques are crucial to determine the PNM classification for a given patient and in each institution should serve as a basis for the multidisciplinary evaluation of each case and common decision on the patient’s care management. This makes ultrasonographers, radiologists, and nuclear medicine specialists key actors for the care management of patients with HAE.

## 5. Innovation and advanced imaging for HAE

More advanced techniques complementary to conventional US, CT, and MRI have been described in the last decade and could be of help to improve diagnosis, indication for treatment, and/or follow-up of patients with HAE. However, until now, such techniques have been used in a limited number of AE referral centers, most often in only one center. Their diagnostic usefulness, compared to other more conventional approaches, has not been fully evaluated; and they cannot be recommended for routine use until such an evaluation has been performed. The following sections will thus be mostly descriptive, in anticipation of further evidence for wider use of the various techniques currently under study.

### 5.1. Innovation in US imaging of HAE

**Figure 7.**
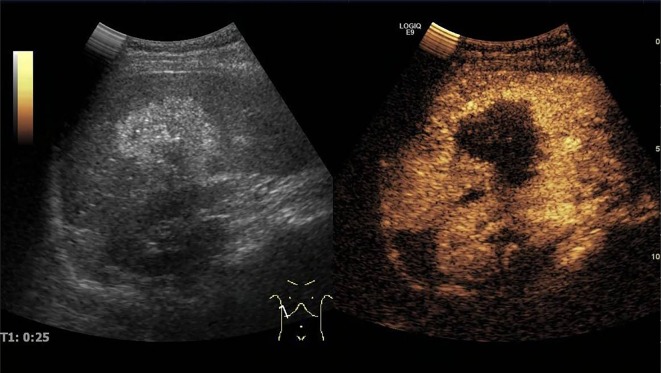
*Contrast-enhanced Ultrasonography (CEUS) using SonoVue*^®^*in a case of hepatic alveolar echinococcosis.* CEUS showed a hepatic mass with irregular rim enhancement in the peripheral areas and no enhancement within the central areas at the hepatic artery phase (25 s).

Micro-bubble contrast agents have been developed to improve US imaging and the technique of “contrast-enhanced ultrasonography” (CEUS) has been proposed for the diagnosis and evaluation of AE lesions. However, the nature of the images and the clinical interest of CEUS in AE were initially controversial. First attempts used contrast-enhanced wide-band Doppler ultrasonography, with Levovist^®^ as a contrast agent: In five patients with AE, Suzuki et al. [41] observed defect patterns with distinct and irregular margins looking like “worm-eaten defects”, and proposed Levovist^®^-CEUS as a clinically useful imaging method for the early diagnosis of AE and improvement in lesion size evaluation. However, using the same technique [26], Kratzer et al. reported that even though Levovist^®^-CEUS could improve the demarcation of the lesion from the surrounding tissue, it could not detect increased vascularization in any of the 15 patients studied. A significant improvement in CEUS has come with the use of SonoVue^®^ as the contrast agent. In 2007, the same German team [17] compared hepatic lesions in patients with confirmed AE using CEUS, three-phase helical CT and FDG-PET and demonstrated that CEUS had higher concordance with FDG-PET in exploring the activity of AE than CT and was even more sensitive than FDG-PET/CT to identify “disease activity”. In their study, FDG-PET identified increased metabolic activity in the lesions in 7 patients (41.2%); a typical vascularization pattern of the lesions was visualized in nine patients (52.9%) by CEUS and in four patients (23.5%) by CT. All positive FDG-PET findings were also positive on CEUS. More recently [42], 17 Chinese patients with 19 AE lesions were examined with “basic US”, color Doppler flow imaging (CDFI), and then CEUS, before any treatment. Examined by basic US, 47.4% of AE lesions showed irregular hyperechoic areas and 52.6% appeared as having a mixed echotype with irregular anechoic areas in the central part of the lesions. CDFI indicated no blood flow signals inside any of the 19 lesions. By CEUS, all 19 lesions displayed a circular rim enhancement in the peripheral areas and no enhancement within the central areas of the lesions (a “black hole” effect) (Fig. 7). As a result, the lesion margins were clear, irregular, and distinct. In general, the sizes of all AE lesions observed by CEUS were larger than those obtained by basic non-contrast-enhanced US. The last two studies strongly suggested that CEUS could bring about significant improvement in the follow-up of AE patients, by decreasing the number of FDG-PET/CT exams performed, thus reducing patient irradiation and the cost of follow-up. However, in all series of cases under study, the number of patients included (5–17 patients) as well as the variety of lesions, according to the usual imaging classifications of AE, were rather limited. Their results should be extended to a larger number of patients before formally recommending CEUS as a standard imaging exam for the follow-up of AE. In addition, the disappearance of the “metabolic activity” of the lesions, as assessed by FDG-PET/CT, which is recommended to decide upon medical treatment withdrawal, is likely different from the information about lesion vascularization given by CEUS. Moreover, the use of CEUS images as a basis for treatment withdrawal has never been reported and correlation with specific antibody disappearance, another surrogate marker of parasite inactivity, has never been studied.

In Xinjiang Medical University hospital, experimental studies have complemented the first clinical experience with CEUS, and they give some clues as to the pathological substratum of CEUS images. Such studies in *E. multilocularis*-infected rats using CEUS have shown that the enhancement of early lesions can generally be divided into two different types [52]. Lesions of a size smaller than 3 mm exhibited ring enhancement during the arterial phase and no enhancement during the portal-venous phase. The larger lesions showed ring and central septa enhancement during the arterial and portal-venous phases. Pathologically, all these lesions were identified as single vesicle- or multiple vesicle-structures with a surrounding inflammatory reaction belt including proliferation of small vessels around the lesions. With *E. multilocularis* further growth, advanced lesions presented the typical ring enhancement at the arterial phase and no enhancement in the center of lesions at the arterial and portal-venous phases. Microscopic examination of the pathological samples identified all the lesions as multiple vesicle-structures mixed with fibrous tissue, surrounded by an inflammatory reaction belt, still including proliferating small vessels around the lesions. Because SonoVue^®^ is a blood pool contrast agent, it may be anticipated that the proliferation of small blood vessels in the inflammatory reaction belt is the basis for the enhancement after injection of the contrast agent. The regular enhancement pattern suggests that the main blood supply of those smaller lesions was mainly the hepatic artery. However, because of the progression of obstruction and subsequent destruction of small arterial vessels, portal vein flow could participate in blood supply of bigger lesions. All the experimental data thus suggest that CEUS can demonstrate the real host-derived micro-vessel perfusion in HAE [52].

### 5.2. Innovation in CT imaging of HAE

Unlike traditional CT imaging which can only reveal the morphology and density of pathological lesions, Energy CT (including Dual Energy CT, DECT, and Spectral CT, SCT) is a new approach for functional imaging which improves material differentiation [30]. Much research has been conducted on Energy CT for the detection of small lesions, positive and differential diagnosis of tumors, treatment efficacy assessment, and prognosis evaluation [18].

**Figure 8.**
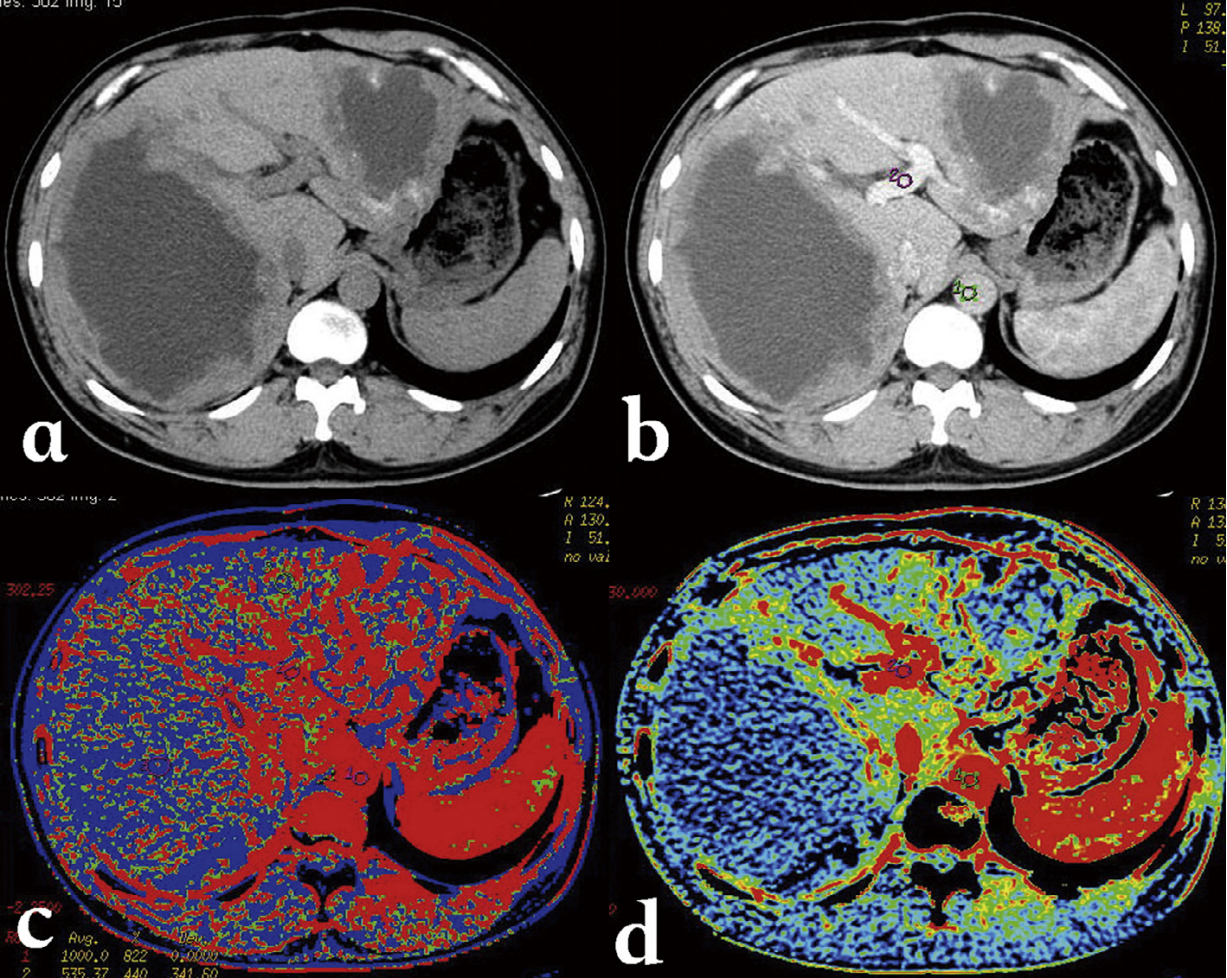
*Alveolar echinococcosis in a 34-year-old man.* (a) CT plain scan shows a cyst-solid mixed hepatic mass involving both lobes of liver with irregular margin and necrosis areas of low density in the center. (b) Post-contrast phase shows a non-enhancing, hypo-attenuating lesion. (c) Blood flow map, and (d) blood volume map, at CT perfusion, show the micro-circulation in the marginal area of AE.

**Figure 9.**
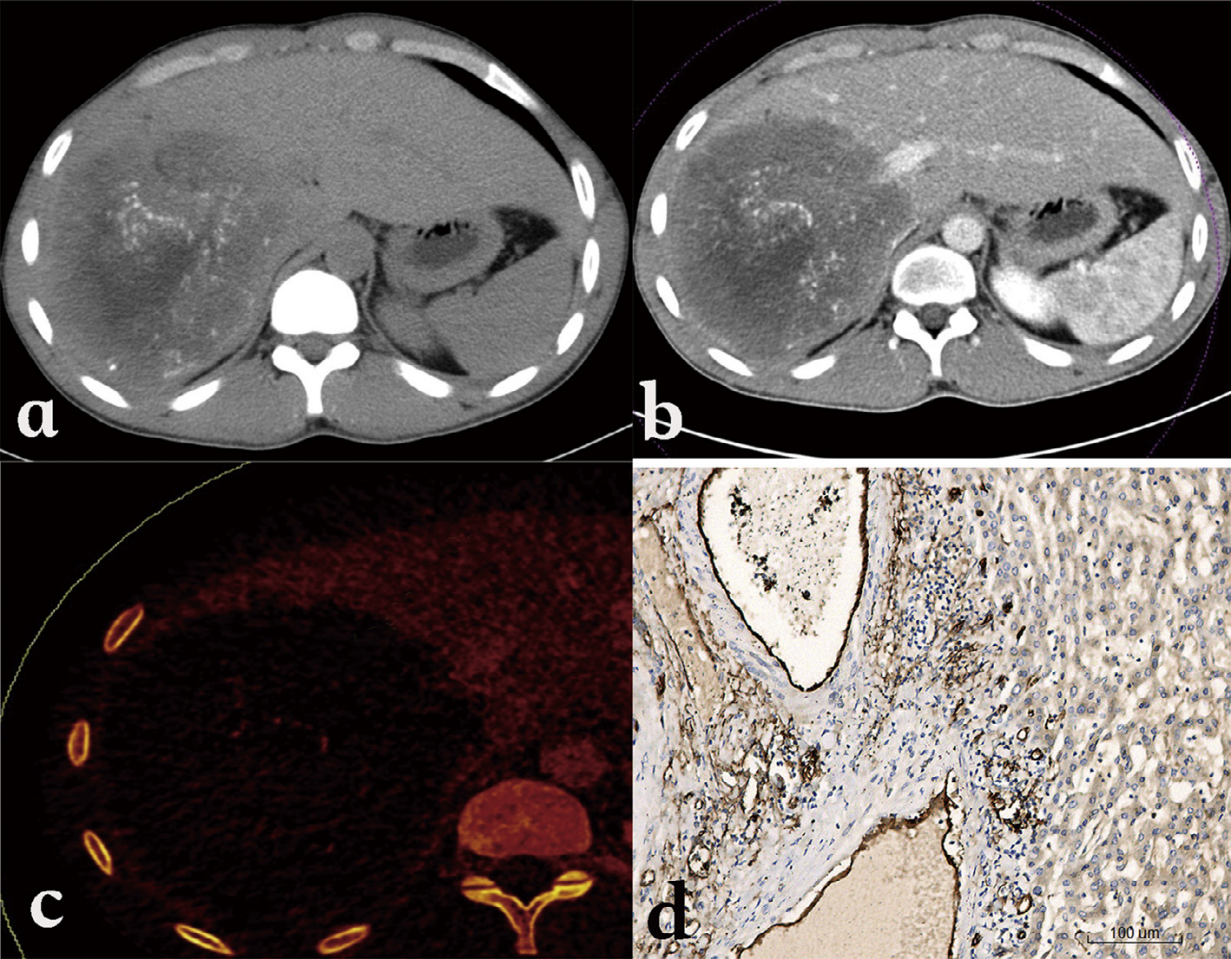
*Alveolar echinococcosis in a 23-year-old woman.* (a) Plain CT shows an infiltrative tumor-like mass with irregular margin and heterogeneous density in the right lobe of the liver. (b) Enhanced CT scan shows mild enhancement at the edge of the mass. (c) Iodine map of Spectral CT shows iodine distribution in the liver and lesion. (d) Micro-vessel density on histopathology of the iodine-enhanced area.

**Figure 10.**
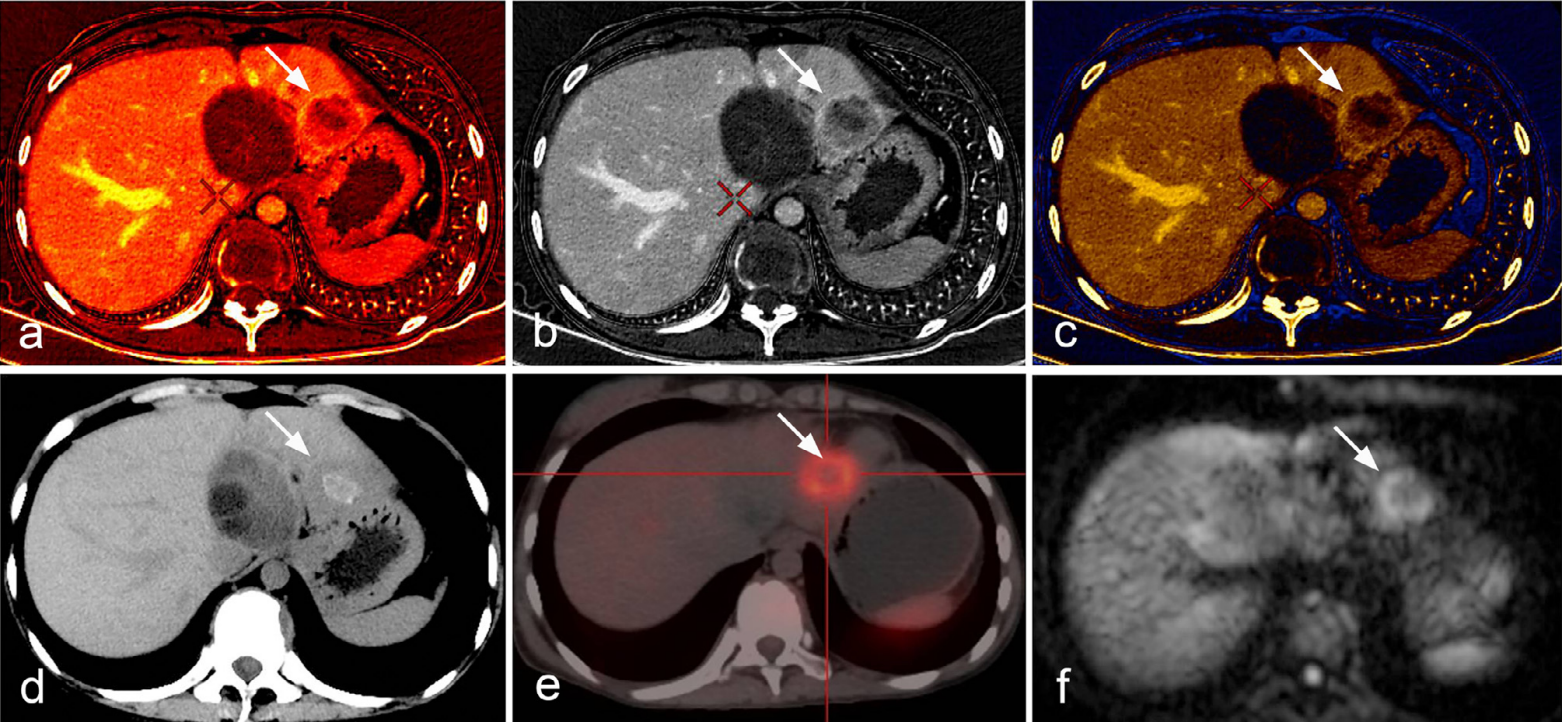
*Comparison between spectral CT, PET-CT and MR-DWI images.* (a, b, c) The iodine map clearly delineates the well-perfused parts of hepatic alveolar echinococcosis in the marginal zone; (d, e) the enhanced and perfused region of AE in spectral CT was consistent with PET-CT image that displayed high uptake, and (f) MR-DWI that displayed high signal.

CT perfusion has been used to detect micro-circulation in HAE lesions, and showed that there was a different level of blood perfusion at the margins of HAE lesions, and a good correlation between blood flow, blood volume and micro-vessel density (MVD) in the same regions of HAE lesions [49] (Fig. 8). However, the high radiation dose of this technique limits its integration into routine clinical practice. Energy CT may more safely demonstrate the same changes in blood supply by measuring parameters of iodine quantification (Kev value, attenuation curves, and iodine concentration) in selected areas of HAE lesions. A study performed at Xinjiang Medical University Hospital in 24 patients showed an excellent correlation between iodine concentration measurements and MVD (*r* = 0.940, *p* < 0.05) in the marginal zone of HAE lesions using DECT (Fig. 9). Iodine concentration in the marginal zone of a HAE lesion was significantly higher than in the solid and cystic components; iodine concentration measurements were significantly different between the marginal zone and the solid component of HAE lesions, both at the artery phase (1.63 mg/mL vs. 0.42 mg/mL, *p* = 0.001) and at the portal-venous phase (1.84 mg/mL vs. 0.62 mg/mL, *p* = 0.001), while mean attenuation values were not significantly different in both phases (55.4 HU vs. 47.2 HU, *p* = 0.063 and 64.4 HU vs. 52.6 HU, *p* = 0.052, respectively). Another study, using SCT, showed that the optimal monoenergetic imaging for HAE was in the level of 65 Kev and different components of HAE lesions could be mapped out by using the different energy attenuation curve: the features of energy spectrum curves of five different patients were similar, although their solid component X-ray absorption values on CT were different. In addition, the fused iodine map clearly delineated the enhanced and well-perfused parts of the AE lesion marginal zone. Such observations probably reflect the neo-vascularization of the periparasitic inflammation developed at the border of the adjacent liver parenchyma. Comparison between results of spectral CT and PET-CT in 14 patients found that the enhanced and well-perfused region of AE in spectral CT was consistent with PET-CT findings (Fig. 10).

These preliminary results indicate that spectral CT imaging based on the spectral differentiation of iodine is technically feasible and can quantitatively identify the micro-perfusion status of the periparasitic granulomatous reaction, and indirectly, as PET imaging does, albeit based on different pathological substratum, reflect the activity of AE lesions. Although they are quite encouraging, these preliminary data should be completed by a prospective systematic study of the information provided by DECT and SCT to assess their cost and risk/benefit value for the functional evaluation of HAE lesions and/or for the follow-up of patients with HAE, compared to the available imaging tools.

### 5.3. Innovation in MRI imaging for HAE

With recent developments in gradient technology, multichannel coils, echo-planar sequences, and parallel imaging techniques, MR-diffusion-weighted imaging (DWI) has become a promising technique for characterizing liver lesions. DWI exploits the microscopic random mobility of water protons to measure the diffusion of fluids in tissue [27]. Tissue regions with restricted water diffusion (e.g. malignant lesions) are displayed as regions of high signal intensity. Signal intensity on DWI can be quantified by calculating the apparent diffusion coefficient (ADC), a valuable indicator for the diagnosis and characterization of focal hepatic lesions [14]. Typically, in HAE the lesions appear hypo-intense on DWI obtained with a b value of <500 s/mm^2^, which results in a higher ADC in the lesion than in the liver parenchyma [15, 38].

At Xinjiang Medical University hospital, we performed preliminary studies on the utility of DWI in detection and characterization of HAE. Our initial study revealed that DWI might improve both the detection and the characterization of HAE lesions. We found a clear advantage of DWI over conventional MR protocols in detecting 59 vs. 52 lesions in patients with HAE; DWI was especially better for detecting small lesions less than 1 cm. In 51 of 59 lesions, with DWI, we observed a peri-lesional higher signal, which was displayed as a continuous or discontinuous circular ring of 2–3 mm thickness, mainly at the lesion’s border with the “normal” liver parenchyma [51]. Such a peri-lesional hyper-signal on DWI could be detected in T2-weighted sequence in only 11 lesions; and it was displayed as only slightly hyper-intense. Because of such impressive results, and as MRI-DW sequences can be easily and quickly performed in clinical practice, even without breathing-control and intravenous contrast injection [14], DWI has already become a routine sequence for HAE detection and evaluation in our center (Figs. 4d and 4e). However, this approach cannot be widely recommended and to gain further diagnostic status, DWI should be prospectively evaluated in other populations of patients, especially in regions like Europe where less advanced HAE cases and HAE cases in immunosuppressed patients are routinely diagnosed.

**Figure 11.**
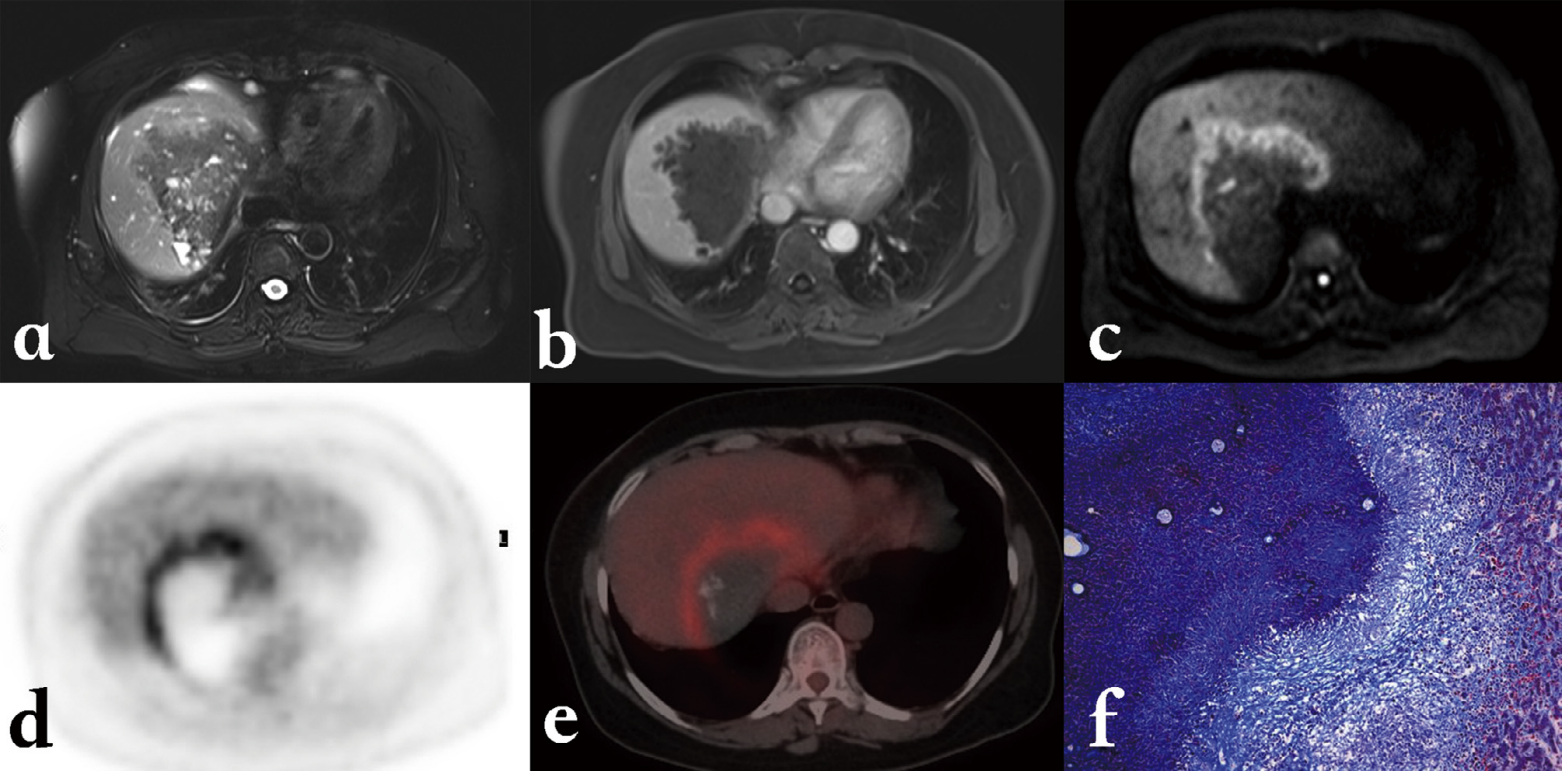
*Alveolar echinococcosis in a 45-year-old patient with hepatic alveolar echinococcosis (HAE).* (a) T2-weighted MR images showed a heterogeneous mass: note the multiple vesicles in the lesion, which showed a well-defined border on enhanced T1WI due to the absence of enhancement of the lesion itself (b). (c) Diffusion-weighted MR images revealed a half circular hyper-intensity area at the lesion’s border with the normal liver parenchyma, which was confirmed to be metabolically active by PET (d) and PET/CT (e). (f) HAE peripheral area was composed of severe fibrosis combined with a large number of inflammatory cells on the histopathological sections (Masson staining; ×100).

We have also analyzed the DWI features of the different components of HAE lesions in 27 cases. Correlations between DWI ADC values and histological markers including micro-vessel density (MVD) and percentage of fibrosis (quantified on pathology sections using Masson staining) were studied. There were significant differences among ADC values in the different areas of HAE lesions. Especially, there was a significant inverse correlation between ADC values and percentage of fibrosis in the peripheral area of HAE lesions (*r* = −0.767, *p* = 0.001). However, there was no significant relationship between ADC values and MVD in the peripheral area (Fig. 11). According to these observations, it appears that ADC values on DWI could represent a potential index of the fibrosis state, but have no value in assessing the peri-lesional micro-circulation of HAE lesions [34, 51]. This fits well with the known physics mechanisms that underpin DWI.

Finally, the potential value of the hyper-intensity zones disclosed using DWI as markers of disease activity was estimated in seven patients by comparing their location with PET/CT images obtained at the same period of time. The location of the peri-lesional hyper-intense areas in HAE lesions on DWI imaging was just similar to the location of the “positive” images in PET/CT imaging. Investigation of more cases is still underway, and the relationship with Kodama types defined on usual T1- and T2-weighted sequences would be worth checking, since hyper-intensity on DWI might also be correlated to the presence of micro-cysts.
